# Simvastatin attenuates chromium-induced nephrotoxicity in rats

**DOI:** 10.15171/jnp.2017.02

**Published:** 2016-08-09

**Authors:** Zahra Goodarzi, Esmaeil Karami, Massumeh Ahmadizadeh

**Affiliations:** ^1^Department of Occupational Health, Engineering, School of Health, Semnan University of Medical Sciences, Semnan, Iran; ^2^Department of Occupational Health, Engineering, School of Health, Ahvaz Jundishapur University of Medical Sciences, Ahvaz, Iran; ^3^Physiology Research Center, Ahvaz Jundishapur University of Medical Sciences, Ahvaz, Iran

**Keywords:** Simvastatin, Sodium dichromate, Malondialdehyde, Glutathione, Nephrotoxicity

## Abstract

**Background:**

Hexavalent Chromium (Cr (VI)) compounds are extremely toxic and have been demonstrated to induce nephrotoxicity associated with oxidative stress in humans and animals. The wide environmental distribution of these agents lead to an increase interest of preventive effects of its adverse effects.

**Objectives:**

The propose of the present study was to determine the potential protective effects of simvastatin (SIMV) on Cr (VI)-induced nephrotoxicity in rat.

**Materials and Methods:**

Forty-eight adult male Wistar rats (180-220 g BW) were randomly assigned to eight groups (n = 6). Group one received SIMV 20 mg/kg/day. Group two was given vehicle only. Groups three, five and seven received intraperitoneally (i.p) sodium dichromate (Cr (VI)) at doses of 8, 12 and 16 mg/kg body weight. Groups four, six and eight pretreated with the 20 mg/kg SIMV 30 minutes to prior administration of Cr (VI) at doses of 8, 12 and 16 mg/kg, respectively. The experiment repeated for eight consecutive days. Twenty-four hours after the last administration, animals were killed with overdose of sodium pentobarbital. Kidney tissues were excised for measuring malondialdehyde (MDA), glutathione (GSH) and histopathological examination.

**Results:**

Chromium induced a dose dependent elevation of MDA and reduction of GSH levels. Histopathological manifestations were observed in Cr (VI)-treated rats. SIMV administration restored Cr (VI) produced biochemical and morphological changes in rat kidney. SIMV decreased MDA values and increased GSH levels in Cr (VI)-treated rats. SIMV clearly reversed the microscopic damage, demonstrating its protective effects against Cr (VI)-induced kidney injury.

**Conclusions:**

This observation suggests that SIMV may have a protective effect against Cr (VI)-induced oxidative stress in rat kidney.

**Implication for health policy/practice/research/medical education:**

In an experimental study, we found that simvastatin (SIMV) as an antioxidant agent protects kidney against chromium induced nephrotoxicity. The mechanism of this renoprotective effects mainly includes amelioration of lipid peroxidation produced by chromium as well as elevation of glutathione (GSH).

## 1. Background

Hexavalent chromium (Cr (VI)) compounds are extremely toxic and carcinogenic. Environmental and occupational exposure to chromium compounds especially hexavalent chromium (Cr (VI)), is widely recognized as a potential nephrotoxic in humans and animals (1-6). Several investigators reported chromium-induced kidney disease in humans (1-3). Renal dysfunction in hard-chrome plating workers are reported (2). It has been suggested that long term exposure to chromium Cr (VI) produced chronic renal injury and caused alterations in renal function among ferrochromium-producing workers (3). Sahu et al found that subcutaneous injection of chromium (VI) resulted in histopathological alterations in the rat kidney (4). Balakrishnan et al reported that administration of chromium in rats significantly reduced the antioxidant markers such as superoxide dismutase and reduced glutathione (GSH) along with significant increase in peroxidation markers such as malondialdehyde (MDA) in the rat kidney as compared with control animals (5). Khan et al exhibited Cr (VI) produced injury in rat kidney (6). Velma and Tchounwou found Cr (VI) induced significant levels of oxidative stress in both liver and kidney organs, however the kidney appears to be more vulnerable and sensitive to Cr-induced toxicity than the liver (7). Molina-Jijón et al found K(2)Cr(2)O(7)-induced renal dysfunction, histological damage, oxidant stress, and the decrease in antioxidant enzyme activity in rat kidney tissue (8) Arreola-Mendoza et al observed that chromium produced kidney injury in female rat (9). Likewise, Parveen et al reported that chromium-treated rats showed significant increases in markers of renal injury in serum, including blood urea nitrogen (BUN), serum creatinine and decreased levels of GSH and catalase activity in the kidney homogenate of Cr (VI)-treated rats. These results were also supported and confirmed with histopathological findings (10).

The mechanism by which Cr (VI) induced nephrotoxicity is not completely understood. However, the role of oxidative stress in injury associated with hexavalent chromium exposure suggests that anti-oxidant supplementation may mitigate chromate-induced toxicity.

Statins are commonly used for lowering serum cholesterol levels (11). In addition, these agents have antioxidant property (12). Simvastatin (SIMV) has been shown to be protective agents against renal injury induced by cisplatin in rat and lead in mice (13,14). However, the effects of this chemical on Cr (VI)-induced nephrotoxicity has not been reported.

## 2. Objectives

The purpose of this study is to investigate the effects of SIMV on Cr (VI)-induced nephrotoxicity in rat.

## 3. Materials and Methods

Our study is a randomized controlled trial, approved by the ethical committee of Jundishapur University of Medical Sciences, Ahvaz, Iran.

### 3.1. Chemicals

All reagents and chemicals were of analytical grade or higher purity. Sodium dichromate was purchased from Aldrich Chemical Co., simvastatin obtained from Tehran Chemie pharmaceutical Co., 1,1,3,3-tetraethoxypropane (TEP) was prepared from Merck Chemical Co., other products included 5,5-dithiobis,2-nitrobinzoicacid (DTNB), trichloroacetic acid (TCA), thiobarbituric acid (TBA), reduced glutathione (GSH) and sodium pentobarbital were supplied from Sigma Chemical Co.

### 3.2. Animal treatments

Adult male Wistar rats (180-220 g) were randomly divided into eight groups of six rats each. The animals were kept in the standard 12-hour darkness, brightness and temperature 23±2°C. The groups were assigned the following regimens: Group one: rats were treated (oral gavages) with SIMV at dose of 20 mg/kg BW/day. Group two: used as controls (given vehicle, normal saline). Groups three, five and seven were received intraperitoneal (ip) sodium dichromate at doses of 8, 12 and 16 mg/kg. Groups four, six and eight were pretreated with SIMV (20 mg/kg body weight, oral gavages) 30 minutes prior to administration of sodium dichromate (8, 12 and 16 mg/kg body weight). All groups were treated for eight consecutive days. Twenty-four hours after the last treatment, all rats were killed with overdose of sodium pentobarbital. Kidney tissues removing were washed with normal saline. The part of kidney tissues were removed, fixed and processed for light microscopy, using hematoxylin-eosin (H&E) staining technique. Five histological sections each at least 15 μm apart were taken from each tissue block and stained with H&E. The criteria for cell injury included nuclear dilation, loss of staining capacity and obvious cellular swelling. Then, other part of kidney tissues were collected for determination of MDA and GSH.

Peroxidation markers: MDA, the product of lipid peroxidation, was estimated by the method described by Buege and Aust (15). Tissues lipid peroxidation was measured in whole-kidney homogenate at 10000 g for 10 minutes, the supernatant was taken. Aliquots (1 mL) were analyzed for MDA content after the addition of 2 mL of TBA reagent. Then tube was in vortex mix for 10 seconds and placed in a boiling water bath (90-100°C) for 20 minutes. After cooling for 7 minutes, the resulting supernatant was removed and measured at wave of 532 nm with the use of the SERIEC-7000 spectrophotometer. MDA concentration was determined by using 1,1, 3, 3-tetraethoxypropane as external standard (0.5-2.5 μM).

Estimation of reduced GSH: Reduced GSH (GSH) was measured by the method of Ellman (16). For measurement of the kidney tissues GSH, 5 mL of aliquots was added to 4 mL distill water (DW) and 1 mL of 5% TCA and also, the mixture was vortexes and centrifuged at 3000 g for 15 minutes. Then 2 mL of supernatant was added to 4 mL Triss buffer (0.4M, pH 8.9) and 0.1 mL of DTNB. The mixture was allowed to stand for 5 minutes, and forming a yellow substance. The absorbance was measured at 412 nm.

### 3.3. Ethical issues

The research was approved by ethical committee of Jundishapur University of Medical Sciences. Prior to the experiment, the protocols were confirmed to be in accor­dance with the guidelines of Animal Ethics Committee of Jundishapur University of Medical Sciences**.**

### 3.4. Statistical analysis

The data were analyzed using SPSS 16.0. Data were analyzed by using One-way analysis of variance (ANOVA), followed by post hoc analysis with LSD test. Probability value of ≤0.05 was determined to be statistically significant.

## 4. Results

**Figure 1:**
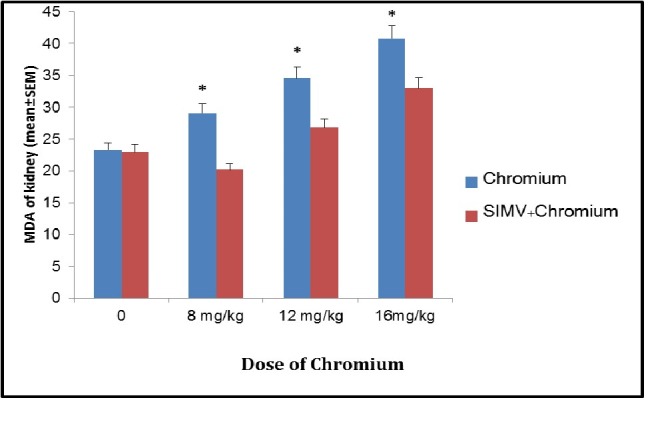
Effects of SIMV on MDA level in rat kidney tissues treated with sodium dichromate (chromium). *Significantly different from rats pretreated with SIMV and were given the same dose of chromium (P ≤ 0.05).

**Figure 2:**
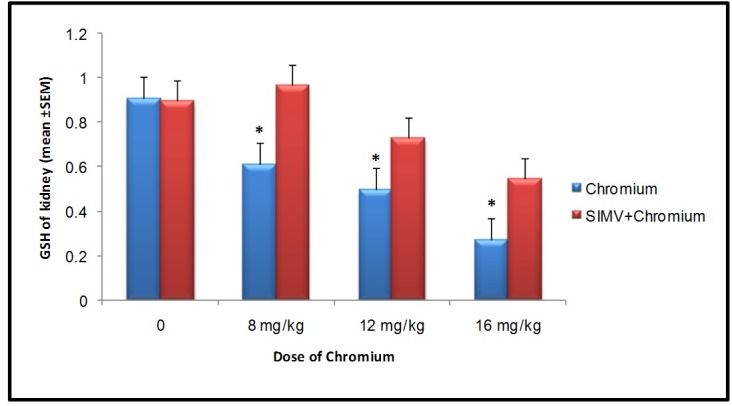
Effects of SIMV on GSH level in rat kidney tissues treated with chromium. *Significantly different from rats pretreated with SIMV and were given the same dose of chromium (*P*≤0.05).

Sodium dichromate (Cr (VI)) induced dose-dependent elevation of MDA level in rat kidney when compared to the control group (*P*≤0.05). SIMV had no effect on MDA levels in rat kidney when compared to those in control value, but this chemical significantly (*P*≤0.05) decreased MDA concentration in Cr (VI) treated rats when compared to those which received the same dose of Cr (VI) only (Figure 1). The level of GSH significantly decreased in Cr (VI) treated rats when compared to control animals. However, pre-treatment of animals with SIMV markedly increased GSH levels in Cr (VI) treated rats when compared to those in non-pretreated rats which received the same dose of Cr (VI) (Figure 2).

**Figure 3:**
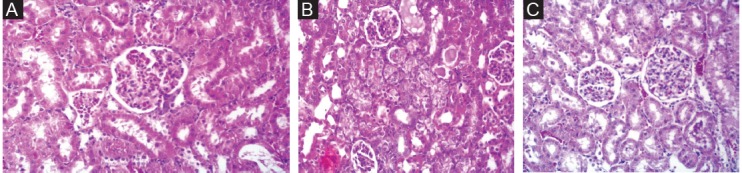
(A) Light micrograph of rat kidney treated with normal saline (control) showing the normal architecture of renal tissue. (H & E 20×). (B) Light micrograph of rat kidney treated with 16 mg/kg Cr (VI) showing tubular necrosis, loss of staining capacity and dilation of proximal convoluted tubular cells (arrow). (H & E 20×). (C) Light micrograph of rat kidney pretreated with 20 mg/kg SIMV 30 min prior to administration of 16mg/kg Cr (VI). Showing no obvious injury in the kidney tissue (H&E 20×).

Administration of saline (vehicle) alone did not produce detectable injury in rat kidney (Figure 3A). However, Cr (VI)-induced damage in the kidney tissue. The loss of the nuclei in the lining epithelium of the necrotic tubules were noted. Loss of the nuclei in the lining epithelium of the necrotic tubules, nuclear dilation, loss of staining capacity and obvious cellular swelling were observed in chromium-treated rats (Figure 3B). The extent of injury was increased in dose dependant manner. SIMV had no obvious injury in rat kidney and the kidney tissue was similar to control animals. However, this agent protected kidney damage in Cr (VI) treated rats (Figure 3C).

## 5. Discussion

Nephrotoxicity is one of the most common adverse effects of toxic chemicals. The results of the present study demonstrated that Cr (VI) produced dose–dependent adverse effects on rat kidney. Nephrotoxicity of Cr (VI) was reported by several investigators (1-5). Our findings along with others suggested that kidney is susceptible for Cr (VI)-induced toxicity. We observed that Cr (VI) produced damage mainly in proximal convoluted tubu­lar cells. Our finding is consistent with previous reports (1,3,17). The proximal convoluted tubular segment represents the most susceptible site of injury via metabolic activation (17). It has been reported that Cr (VI) compounds are selectively accumulated in the proximal convoluted tubular cells where in high dose induce renal tubular injury (1,3,17). We observed that Cr (VI) nephrotoxicity was accompanied by decreased renal GSH. Reduced GSH is considered to play a central role in protection of cells from oxidant injury. We found that the depletion of GSH in a dose dependent manner in chromium treated rats. Standeven et al showed that GSH protects against the acute nephrotoxicity of chromium (18). We also found that the exposure of rats to Na (2) Cr(2)O(7) for 8 consecutive days provoked renal damages with a significant increase in kidney MDA. Accordingly, Yonar et al reported that the levels of MDA, as an index of lipid peroxidation, increased as well as reduced GSH levels in Cyprinus carpio kidney treated with chromium (19).

The mechanism by which Cr (VI) produced renal injury was not completely understood. However, Gunaratnam et al reported that pretreatment with cytochrome p450 inducing agents, either phenobarbitone (PB) or 3-methylcholanthrene (3-MC) resulted in amelioration of Cr (VI) toxicity in rat liver. They suggested that the inducing agents increase the amount of enzymatic reduction of Cr (VI) (20).

Since kidneys usually have low drug-metabolizing en­zyme activities, chemically induced nephrotoxicity has been assumed to be produced by toxic intermediate(s) generated in the liver and transported to the kidney. As Cr (VI) is eliminated via the kidney, the generation of me­tabolites in kidney may at least in part be responsible for kidney toxicity. Another possibility for chromium caused renal damage is that translocation of Cr (VI) metabo­lites from the liver to the kidney via general circulation produced kidney injury.

We found that pretreatment of animals with SIMV markedly decreased lipid peroxidation and increased GSH levels in Cr (VI) treated rats. These findings suggested that SIMV protected kidney cells against chromium toxicity and support the view that these organs have ability to metabolize Cr (VI) and induced oxida­tive stress. Parihar et al found that SIMV lower calcium-induced oxidative stress in rat isolated mitochondria (21). Additionally, Yao et al observed that SIMV attenuates MDA level and protects the heart against myocardial injury caused by acute myocardial ischemia (22).

The present study was consistent with previous studies in finding that SIMV may attenuate renal injury induced by Cr (VI). Furthermore, Iseri et al found that SIMV attenuates cisplatin –induced renal damage (13). Mohammadi et al found that pretreatment of rats with SIMV caused protective effects on renal tissue of mice expose to lead (14). Moreover, Todorovic et al showed that pretreatment with SIMV significantly protected rat kidney injured by ischemia-reperfusion injury (12). On the basis of these results, we conclude that SIMV may also prevent the occurrence of chromium VI-induced adverse effects in kidney.

## 6. Conclusions

In conclusion, Cr (VI) caused biochemical and structural alterations in rat kidney. Administration of SIMV improved biochemical and histopathological alterations induced by chromium in rat. It seems SIMV due to antioxidant property has protective effect on kidney and thereby reduced generation of reactive toxic metabolites responsible for chromium–induced nephrotoxicity.

## Acknowledgements

The authors wish to thank the research deputy of Ahvaz Jundishapur University of Medical Sciences for offering the grants for this investigation. The source of data used in this paper was from master thesis of Zahra Goodarzi, student of Occupational Health Engineering Department, School of Health, Ahvaz Jundishapur University of Medical Sciences, Ahvaz, Iran. Our special thanks go to Dr. B. Mohammadian for reviewing histopathologi­cal samples.

## Authors’ contribution

MA designed the research. ZG and EK provided extensive intellectual contribution, collection and analysis of the data and wrote some parts of paper. MA prepared the final draft.

## Conflicts of interest

The authors declared no competing interests.

## Funding/Support

This study was supported by Physiology Research Center and the research deputy of Ahvaz Jundishapur University of medical sciences (Grant NO. PRC-151).
